# To-be-forgotten information shows more relative forgetting over time than to-be-remembered information

**DOI:** 10.3758/s13423-023-02330-1

**Published:** 2023-07-11

**Authors:** Anna T. Nickl, Karl-Heinz T. Bäuml

**Affiliations:** Department of Experimental Psychology, Regensburg University, 93040 Regensburg, Germany

**Keywords:** Episodic memory, Forgetting, Directed forgetting, Time-dependent forgetting

## Abstract

People can intentionally forget studied material when cued to do so. Corresponding evidence has arisen from studies on item-method directed forgetting, in which participants are asked to forget single items directly upon presentation. We measured memory performance of to-be-remembered (TBR) and to-be-forgotten (TBF) items across retention intervals of up to 1 week and fitted power functions of time to the observed recall (Experiment 1) and recognition (Experiment 2) rates. In both experiments and each retention interval condition, memory performance for the TBR items was higher than for the TBF items, supporting the view that directed forgetting effects are lasting. Recall and recognition rates of both TBR and TBF items were well fit by the power function. However, the relative forgetting rates of the two item types differed, with a higher forgetting rate for the TBF than the TBR items. The findings are consistent with the view that TBR and TBF items differ (mainly) in recruitment of rehearsal processes and resulting memory strength.

## Introduction

Every day we are faced with a staggering array of new information and changing demands. Some of this information needs to be permanently stored in memory while other matters become irrelevant and should be discarded. This necessitates mechanisms for memory updating – processes by which stored contents are flagged as relevant or irrelevant depending on current and future demands, and then handled accordingly. Memory research suggests several such possible mechanisms, like, for instance, the calculation of future relevance based on prior use (Anderson & Schooler, 1991).

Updating can also be induced by cues that convey the relevance of information, presented during or shortly after the acquisition of material. A prominent example is directed forgetting, which encompasses instructions to remember or forget studied material (for reviews, see Anderson & Hanslmayr, 2014; MacLeod, 1998; Sahakyan et al., 2013). In item-method directed forgetting (IMDF), subjects study a list of items and, after study of each single item, receive either a remember or a forget cue, providing information on whether the particular item will later be tested, or not. Within the study list, the two types of items are randomly intermixed for each participant. Results show that, on a final test for all studied items, memory for the to-be-forgotten (TBF) information is typically worse than is memory for the to-be-remembered (TBR) information, which defines the IMDF effect.

The two most prominent theoretical accounts of IMDF – the selective rehearsal and the inhibition accounts – both stress differences in encoding of TBR versus TBF information. The selective rehearsal account assumes that items are maintained in memory until the presentation of a forget or a remember cue, upon which TBF items are dropped from further rehearsal, while rehearsal for TBR items continues (Basden et al., 1993; Bjork, 1970). In contrast, the inhibition account assumes that encoding of TBF items is impaired through memory control processes that downregulate these items’ memory representations (Fawcett & Taylor, 2008; Fellner et al., 2020; Rizio & Dennis, 2013). This line of work sees forgetting as a more active process, contrary to the assumption of a merely passive dropping of TBF items from further rehearsal. Inhibition has also been argued to operate in concert with selective rehearsal processes (Anderson & Hanslmayr, 2014; Fellner et al., 2020).

Most IMDF studies in the literature employed a relatively short retention interval of a few minutes between study and test (see MacLeod, 1998), but there is also evidence that the effect can persist after a prolonged retention interval. Indeed, a few studies reported IMDF effects after delay intervals of 90 min, 6 h, or 1 week with free-recall testing (Basden & Basden, 1998; Saletin et al., 2011; Scullin et al., 2017) and after delay intervals of 1 or 2 weeks with item-recognition testing (MacLeod, 1975, 1989). The studies, however, are largely silent on whether TBR and TBF information differ in forgetting rates as time after study passes, as rates of time-dependent forgetting of the two types of information have not been analyzed yet.

Memory typically declines rapidly soon after encoding followed by a long, much slower decline in memory performance (Ebbinghaus, 1885). This curvilinear nature of time-dependent forgetting has been well captured by a power function of time, *r*(*t*) = *at*^−*b*^, where *r*(*t*) represents proportion of recalled items at time *t*, parameter *a* represents recall level after one unit of time, and parameter *b* represents relative forgetting (forgetting rate) as time passes.^1^ While the power function is able to describe time-dependent forgetting over a wide range of experimental situations (Rubin & Wenzel, 1996; Squire, 1989; Wixted & Ebbesen, 1991, 1997), time-dependent forgetting of TBF information has not been studied yet. It is therefore unclear whether the power function can also capture time-dependent forgetting of TBF information, and, if so, whether TBR and TBF information show similar or different forgetting rates – i.e., similar or different forgetting rate parameters *b* – as time after study passes.

Current accounts of IMDF make no clearcut predictions on whether TBR and TBF items should differ in forgetting over time. Some expectations, however, may be formulated if findings from related paradigms are taken into account. Wixted (2022) recently fitted power functions of time to recall data from several studies, in each of which, for a number of retention intervals, recall of items with a high degree of learning was compared to recall of items with a low degree of learning – high and low degrees of learning were implemented by different numbers of study trials. For these studies, Wixted (2022) found that a high degree of learning is accompanied by a lower relative rate of forgetting – i.e., a smaller forgetting rate parameter *b* – than a low degree of learning. If (mainly) selective rehearsal mediated IMDF and the remember and forget cues thus created stronger TBR and weaker TBF items, the two item types may well differ in forgetting over time and TBF information show higher relative forgetting – i.e., a larger forgetting rate parameter *b* – than TBR information.

In contrast, TBF information may show a lower forgetting rate than TBR information if (mainly) inhibition mediated IMDF. This expectation arises because inhibitory effects – as they can for instance be observed in retrieval-induced forgetting, the demonstration that selective retrieval practice on some studied items can cause forgetting of the unpracticed items (e.g., Anderson et al., 1994) – have often been found to dissipate with delay (Abel & Bäuml, 2014; MacLeod & Macrae, 2001; see also Bäuml & Kliegl, 2017), which lowers the forgetting rate of inhibited items. The same expectation also emerges from the more general view that inhibition should only temporarily reduce the accessibility of affected items (Bjork, 1989). Thus, depending on whether (mainly) selective rehearsal or (mainly) inhibition mediated IMDF, the forgetting rate of TBF items may be larger or smaller than the forgetting rate of TBR items.

This study addresses the issue and examines time-dependent forgetting of TBR and TBF information. In each of two experiments, subjects studied a list of items, individually followed by a cue to remember or forget the item for an upcoming memory test. The same four retention intervals between study and test (3 min, 1 day, 3 days, 1 week) were employed in the two experiments. At test, memory was measured for all studied items, using a free-recall format in Experiment 1 and an item-recognition format in Experiment 2. We examined in the first step whether not only time-dependent forgetting of TBR information but also time-dependent forgetting of TBF information follows a power function of time. If so, in the second step, we compared forgetting rates – i.e., forgetting rate parameter *b* of the power function – between the two item types, which provides information on whether (mainly) selective retrieval or (mainly) inhibition mediate this form of directed forgetting.

## Experiment 1

### Method

#### Ethical considerations

All reported studies were carried out in accordance with the provisions of the World Medical Association Declaration of Helsinki.

### Participants

One hundred and twenty participants took part in the experiment (*M* = 24.12 years, range = 18–30 years; 86 female). They were recruited mainly from Regensburg University, as well as by placing online advertisements in students’ groups in Germany. Eighty percent of the participants were currently enrolled at university, while the remaining subjects reported being employed. Participants were distributed equally across the four between-subjects conditions, yielding n = 30 participants in each condition. Previous work on IMDF and on time-dependent forgetting mostly reported large effects of IMDF and time-dependent forgetting (*d >* 0*.*80; e.g., Kliegl et al., 2019; MacLeod, 1975; Scullin et al., 2017). Sample size in the present experiments was therefore determined on the basis of a power analysis with the G*Power program (version 3.1.9.7; Faul et al., 2009) using alpha = 0.05 and beta = 0.20 as well as effect sizes of d = 0.80 for expected time-dependent forgetting and expected IMDF. Participants were compensated either with a small monetary amount or with partial course credit.

### Materials

Twenty concrete, unrelated nouns (four to six characters) were drawn from the CELEX database, using Wordgen v1.0 Software Toolbox (Duyck et al., 2004) and divided into two sets of ten words each. The items of each set were all paired with either an instruction to forget or an instruction to remember them for an upcoming test. Assignment of sets to instructions was counterbalanced across participants. Study materials as well as data for both experiments can be found on the Open Science Framework (https://osf.io/j8myp/).

### Design

We conducted a 2 (CUE: forget vs. remember) × 4 (DELAY: 3 min vs. 1 day vs. 3 days vs. 7 days) mixed factorial design. CUE was manipulated as a within-subject factor, whereas DELAY was varied between subjects. In each delay condition, the assignment of the item sets to either the forget or the remember instruction was counterbalanced across participants.

### Procedure

Data collection took place via Zoom meetings (Zoom Video Communications), in which subjects and experimenters were connected by live web-cam and microphone feeds. For participants in the 3-min delay condition, the experiment took place during a single session, while for all other participants two Zoom meetings were held with 1, 3, or 7 days between them. The second meeting was always scheduled for the same time of day as the first one (± 2 h). During each session, the experimenter shared their screen and instructed subjects orally. The software OpenSesame (version 3.3, Mathôt et al., 2012) was used for stimulus presentation and balancing.

For all participants, the experiment started with the study phase, during which all 20 items were presented individually in the middle of the screen (4 s). Each item was followed either by the instruction “FORGET” in red font or “REMEMBER” in green font (1 s). Presentation order was pseudo-randomized with each cue type presented no more than three times in succession. Subjects were informed beforehand that only items followed by “REMEMBER” would be relevant for an upcoming test at the end of the experiment and that items followed by “FORGET” could be discarded from memory. Following the study phase, all participants, except for those in the 3-min delay condition, were asked to count backwards in steps of seven for 2 min as a recency control to hamper active rehearsal of materials during the delay. They were then dismissed and asked to return to their second scheduled meeting 1, 3, or 7 days later. The second meeting began with a 3-min distractor task of solving Raven’s Standard Progressive Matrices (Raven et al., 2000). The task was self-paced and subjects gave their answers orally. The participants in the 3-min delay condition immediately proceeded to this task after the study phase. Finally, all participants were given a 4-min free-recall test for all items that had been presented during study, regardless of cue. Subjects typed their answers into the Zoom chat, one word at a time and in any order they chose. Afterwards, subjects were debriefed, thanked, and compensated.

### Fitting the power function to the recall rates

Using maximum likelihood methods (Riefer & Batchelder, 1988; Wickens, 1982), a power function of time, *r*(*t*) = *at*^−*b*^, was fitted to the recall rates of the TBR and TBF items using group average data (see Bäuml & Trißl, 2022; Trißl & Bäuml, 2022).^2^ To test whether the power function captured the time-dependent forgetting adequately, we compared its goodness-of-fit to the goodness-of-fit of a statistical baseline model, which describes the recall rates of an item type – TBR or TBF items – for *n* delay conditions as the product of *n* independent binomial distributions. The comparison of the two models is based on a likelihood ratio, resulting in an approximate *χ*^2^-test with *n* − 2 degrees of freedom (Riefer & Batchelder, 1988; Wickens, 1982). The parameters of the power function were estimated by maximizing the likelihood ratio. Time was measured in days since the end of the study phase.

We next examined whether parameters *a* and *b* of the power function varied between item types. For this, we combined the data sets of the two types of items and compared the goodness-of-fit of a general power function model – which allows for separate power functions for the two types of items and thereby for two distinct *a* parameters and two distinct *b* parameters – to that of a restricted power function model in which either a common *a* parameter or a common *b* parameter was estimated for the two types of information (Bäuml & Trißl, 2022; Riefer & Batchelder, 1988; Wickens, 1982). The comparison between the two models was again based on the calculation of a likelihood ratio and an ensuing *χ*^2^-test with one degree of freedom. All fitting procedures were written in R (R Core Team, 2021) and implemented in R Studio (RStudio Team, 2020), using optim() from the R package *stats* (version 4.1.1) with a Nelder-Mead method for maximization. All other analyses were carried out in IBM SPSS Statistics for Windows, Version 26.0 (IBM Corp., Armonk, NY, USA).

## Results

**Fig. 1:**
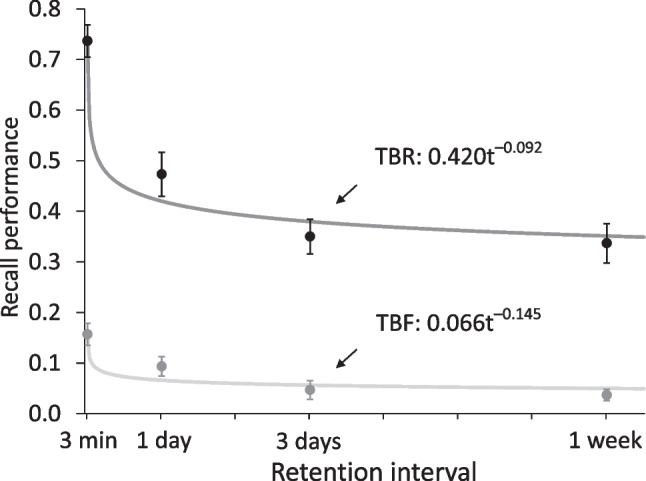
*Results of Experiment 1. *In all four retention interval conditions, recall of to-be-remembered (TBR) items was higher than recall of to-be-forgotten (TBF) items. Recall of both the TBR items and the TBF items showed time-dependent forgetting, described by a power function of time. The TBF items showed a higher relative rate of forgetting, reflected in a larger forgetting rate parameter, than the TBR items. Error bars represent ± 1 standard error

Figure 1 shows recall rates for both TBR and TBF items at all four delay intervals. A 2 × 4 mixed-factors ANOVA with the within-subject factor of CUE (forget, remember) and the between-subjects factor of DELAY (3 min, 1 day, 3 days, 7 days) showed a main effect of CUE, *F*(1*,*116) = 375*.*29, *MSE* = *.*02, *p < .*001, η_*p*_^2^ = 0*.*76, indicating higher recall for TBR than TBF items, and a main effect of DELAY, *F*(3*,*116) = 31*.*72, *MSE* = *.*03, *p < .*001, η_*p*_^2^ = 0*.*45, reflecting time-dependent forgetting. Additionally, there was a significant interaction between the two factors, *F*(3*,*116) = 10*.*61, *MSE* = *.*02, *p < .*001, η_*p*_^2^ = 0*.*22, suggesting a decrease in the size of the IMDF effect with delay. Consistently, follow-up paired *t*-tests between TBR and TBF items demonstrated significant IMDF at all four delay intervals, all *ts*(29) *>* 7*.*59, *ps < .*001, *ds* ≥ 1*.*39, with effect size *d* decreasing with increasing delay (from *d* = 2*.*39 after 3 min to *d* = 1*.*39 after 1 week).

The power function described the time-dependent forgetting of the TBR and TBF items well, as reflected by the *χ*^2^(2)-values of 4*.*90 for the TBR information and 5*.*03 for the TBF information. Indeed, the function accounted for most of the variance in the data, represented by *r*^2^ = *.*96 for the TBR information and *r*^2^ = *.*88 for the TBF information. A direct comparison of the function’s parameters *a* and *b* between item types revealed that parameter *a* differed between item types, and was much higher for the TBR than the TBF items, *χ*^2^(1) = 388*.*89. Importantly, forgetting rate parameter *b* also differed between the two item types, and indicated a higher relative rate of forgetting of the TBF items, *χ*^2^(1) = 3*.*92. TBF items thus showed lower recall shortly after study accompanied by subsequent enhanced time-dependent forgetting.

## Discussion

Experiment 1 covered retention intervals of up to 1 week and is the first experiment in the literature measuring IMDF for more than two delay intervals. Two findings stand out. First, the results show persistent IMDF for all three longer delay intervals (1 day, 3 days, 1 week), which is consistent with the few prior observations of a lasting IMDF effect with free-recall testing (e.g., Basden & Basden, 1998; Scullin et al., 2017). Second, recall rates of the TBR and TBF items revealed typical time-dependent forgetting with recall of both item types following the power function of time. Importantly, TBR and TBF items differed in forgetting rates, with a larger forgetting rate parameter *b* for the TBF than the TBR items, suggesting increased time-dependent forgetting for the TBF items.

Experiment 2 aimed to examine whether the results of Experiment 1 generalize from free-recall to item-recognition testing. In free recall, participants tend to recall items of higher strength before items of lower strength (Wixted et al., 1997), thus potentially inducing output interference and impaired recall on the weaker items. If this finding generalized to (stronger) TBR and (weaker) TBF items and participants selectively rehearsed the TBR information also during the delays (MacLeod et al., 2003), then the prioritization of the TBR items at test might increase with length of the delay interval, leading to an enhanced forgetting rate for the TBF relative to the TBR items. If such recall dynamics underlay the difference in forgetting rates observed in Experiment 1, then the difference should disappear with item recognition, in which testing order is experimenter-guided and random. Item recognition tests with their typically higher memory performance can also circumvent possible floor effects, which might have been present in the recall of TBF items in Experiment 1.^3^

## Experiment 2

### Method

#### Participants

Another 120 participants were recruited (*M* = 22.26 years, range = 18–30 years; 90 female), again mainly from Regensburg University but also by online advertisements. Of the sample, 92.5% were currently enrolled at university; all other participants were either employed or doing vocational training. Participants were again distributed equally across the four between-subjects delay conditions, yielding n = 30 participants in each condition. Sample size followed Experiment 1. Participants were compensated either with a small monetary amount or with partial course credit.

### Materials

Adding to the 20 nouns used in Experiment 1, another 60 concrete, unrelated nouns (four to seven characters) were drawn from the CELEX database, again using Wordgen v1.0 Software Toolbox (Duyck et al., 2004). 40 of these words were used during study, the remaining 40 were used as lures during the final test. The 40 study items (20 words from Experiment 1 and 20 new words) were split into two sets of 20 words each, which were paired with either an instruction to forget or an instruction to remember them for an upcoming test. Assignment of word sets to type of instruction was counterbalanced across participants.

### Design

Like in Experiment 1, we conducted a 2 (CUE: forget vs. remember) × 4 (DELAY: 3 min vs. 1 day vs. 3 days vs. 7 days) mixed-factorial design. CUE served as a within-subject factor, whereas DELAY was varied between subjects. In each delay condition, the assignment of item sets to instructions was counterbalanced across participants.

### Procedure

Again, data collection took place via Zoom meetings. The experiment was identical to Experiment 1 except for the following changes: (a) During study, items were presented for 1.5 s each to avoid ceiling effects, while cues were shown for 1 s as in Experiment 1. Presentation order was again pseudo-randomized, with each cue type presented no more than three times in succession. (b) At test, study and lure items were shown individually for 5 s. Subjects were asked to respond orally with “old” for words they thought they had seen during study, and with “new” for words they thought were new. The experimenter recorded all responses by pressing corresponding keys on their keyboard. Old and new words were intermixed pseudo-randomly, with a maximum of three old or three new words presented in succession.

## Results

**Fig. 2:**
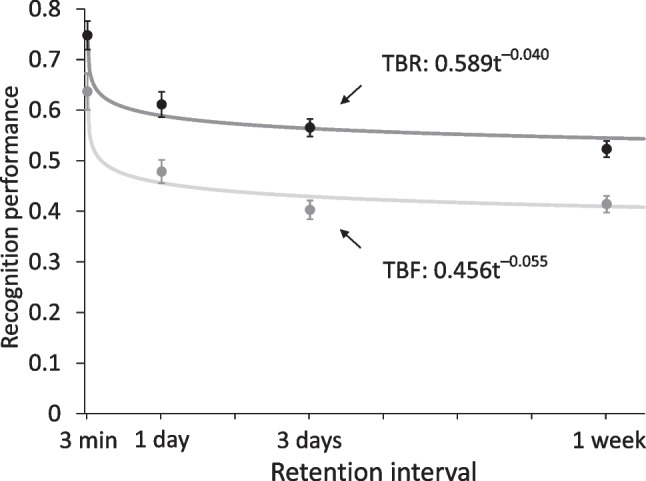
*Results of Experiment 2.* Corrected hits are displayed. In all four retention interval conditions, recognition of to-be-remembered (TBR) items was higher than recognition of to-be-forgotten (TBF) items. Recognition of both the TBR items and the TBF items showed time-dependent forgetting, described by a power function of time. The TBF items showed a higher relative rate of forgetting, reflected in a larger forgetting rate parameter, than the TBR items. Error bars represent ± 1 standard error

Mean false alarm rates (“old” responses to new items) differed between delay conditions, increasing from *M* = *.*16 (*SD* = *.*11) in the 3-min condition to *M* = *.*26 (*SD* = *.*13) after 1 day, *M* = *.*28 (*SD* = *.*11) after 3 days, and *M* = *.*33 (*SD* = *.*11) after 7 days. A univariate ANOVA showed that this increase was significant, *F*(3,116) = 11.84, *MSE* = .01, *p* < .001, η_*p*_^2^ = 0.23, indicating a change in response criterion across delay conditions. Following Wixted and Ebbesen (1991), we therefore corrected the raw hit rates for each cue condition by dividing them by the sum of hit and false alarm rates. Figure 2 shows the corrected hits. A 2 × 4 mixed-factors ANOVA for these corrected hits with the within-subject factor of CUE (forget, remember) and the between-subjects factor of DELAY (3 min, 1 day, 3 days, 7 days) showed a main effect of CUE, *F*(1*,*116) = 211*.*86, *MSE < .*01, *p < .*001, η_*p*_^2^ = 0*.*65, reflecting typical IMDF, and a main effect of DELAY, *F*(3*,*116) = 21*.*84, *MSE* = *.*03, *p < .*001, *η*_*p*_^2^ = 0*.*36, indicating time-dependent forgetting. There was no significant interaction between the two factors, *F*(3*,*116) = 1.99, *MSE* < *.*01, *p = .*119, *η*_*p*_^2^ = 0*.*05.

Next, the power function was fit to the corrected hit rates of the TBR and TBF items. The function described the time-dependent forgetting of the two item types well, as is reflected by the *χ*^2^(2)-values of 2*.*39 for the TBR information and 3*.*06 for the TBF information. Like in Experiment 1, the function accounted for a large part of the variance in the data, represented by *r*^2^ = *.*96 for both the TBR and the TBF items. A direct comparison of the function’s parameters *a* and *b* between the two item types again revealed that parameter *a* differed between item types, *χ*^2^(1) = 81*.*42, with the TBR items showing a higher value than the TBF items. Likewise, forgetting rate parameter *b* did also vary between item types, *χ*^2^(1) = 4*.*50, again with a higher parameter value for the TBF than the TBR items. Thus, like in Experiment 1, TBF items showed lower memory performance shortly after study accompanied by subsequent enhanced time-dependent forgetting.^4^

## Discussion

The results of Experiment 2 replicate those of Experiment 1 using item-recognition rather than free-recall testing. Experiment 2 again found an IMDF effect for all four retention interval conditions, which is consistent with prior recognition work by MacLeod (1975, 1989), who also found IMDF to be lasting. Analogous to Experiment 1, the corrected hit rates revealed typical time-dependent forgetting for both the TBR and the TBF items, with the memory rates of both item types following a power function of time. Critically, like in Experiment 1, the TBR and TBF items differed in time-dependent forgetting, with the TBF items showing a larger relative rate of forgetting – i.e., a larger forgetting rate parameter *b* – than the TBR items. Thus, like the results of Experiment 1, the results of Experiment 2 suggest increased time-dependent forgetting for the TBF items.

## Additional analyses

In prior work, often non-linear least squares were used to estimate power function parameters and, as a descriptive measure, explained variance was reported instead of statistical tests of fit (e.g., Anderson & Tweney, 1997; Rubin & Wenzel, 1996; Wixted & Ebbesen, 1991). We therefore reanalyzed the data of the two experiments using non-linear least squares and calculating explained variance. Additionally, we included d’ as an alternative measure of recognition performance in Experiment 2. The pattern of results obtained with non-linear least squares was almost identical to that found with the maximum likelihood method, regarding both parameter estimates and explained variance (see Table 1). This holds while the parameter estimates for d’ obtained in Experiment 2 naturally differed numerically from those reported above. The findings reported above therefore do not depend on whether maximum likelihood or least-squares methods are employed to estimate parameters and do not depend on exactly which method is used to correct hit rates in item recognition.

**Table 1:** Least squares parameter estimates and explained variance

Condition	a	b	R^2^
*Experiment 1*			
TBR	.422	.091	.962
TBF	.067	.139	.887
*Experiment 2 – corrected hits (H/(H + FA))*			
TBR	.588	.040	.964
TBF	.455	.055	.963
*Experiment 2 – corrected hits (d’)*			
TBR	1.300	.089	.981
TBF	0.436	.140	.943

*Note.* For the calculation of d’, we followed the correction suggested by Macmillan and Creelman (2005) for hit rates of 1 and false alarm rates of 0

## General discussion

The results of this study show that IMDF effects are lasting, in both free recall and item recognition, thereby replicating and extending results from prior work (e.g., MacLeod, 1975, 1989; Scullin et al., 2017). In addition, two results emerge. First, both TBR and TBF information show typical time-dependent forgetting with memory performance declining rapidly soon after study followed by a long, much slower decline in memory performance. Importantly, for both types of information, this decline is well described by a power function of time. Second, both when using free-recall and when using item-recognition testing, forgetting rates differ between the two types of information, with a higher relative rate of forgetting for the TBF information.

The observed persistence of IMDF is in line with the selective rehearsal account of IMDF, as the suggested difference in encoding between TBR and TBF information should translate into long-lasting differences in memory performance. Our finding of different forgetting rates for the two types of information also fits with this account, as the putative difference in encoding should create memories of stronger (TBR) versus weaker (TBF) representations, and memories with stronger representations have been found to show less relative forgetting over time than memories with weaker representations (Wixted, 2022). In contrast, the observed difference in forgetting rates disagrees with the inhibition account, as inhibitory effects are expected to dissipate over time (Bäuml & Kliegl, 2017; Bjork, 1989) and the forgetting rate for the TBF may therefore be reduced relative to the TBR items. The findings, however, do not exclude the possibility that both selective rehearsal and inhibition contributed to forgetting (e.g., Fellner et al., 2020). Critically, in this case, the contribution of selective rehearsal should have been much larger than that of inhibition. Indeed, if the two processes contributed similarly to forgetting, forgetting rates may be comparable between item types; if mainly inhibition contributed, the forgetting rate of the TBF items may be reduced relative to the TBR items – which is not what the results show.

In explaining the difference in forgetting rates between stronger and weaker items, Wixted (2022) speculated that degree of learning might serve as a proxy of how subjectively meaningful studied material is and material of higher meaningfulness, to some degree, may be prevented from forgetting over time, for instance, by prioritizing consolidation of this information (Cowan et al., 2021; Stickgold & Walker, 2013). If TBR items mimicked items of a high degree of learning, TBR items might also benefit from prioritized consolidation and thus, to some degree, be prevented from forgetting over time. Studies on the role of sleep-associated memory consolidation for IMDF effects are consistent with this idea. Examining how a 100-min nap during a 6-h delay between study and recall influences recall of TBR and TBF items, Saletin et al. (2011) found a larger difference between TBR and TBF items after the nap than in a no-nap baseline condition. Critically, the larger difference was due to a selective increase in recall for the TBR items, pointing to overall better consolidation for TBR than TBF information (see also Rauchs et al., 2011).

Prioritized consolidation of TBR items might also arise because of the explicit cue that is provided to the participant about the information’s future need. Indeed, forget and remember cues may elicit different expectations regarding the future relevance of studied material, and thereby influence further processing. This idea is in line with observations of selective sleep benefits for information that is cued as relevant, for instance, by manipulating test expectancy. Using retention intervals of 9 h between study and test that participants either spent awake or asleep, Wilhelm et al. (2011) examined how the information for the participants that there will be a memory test at the end of the retention interval influenced recall after the delay. Memory performance was higher after sleep compared to wakefulness, but only if participants had been told to expect the test. Thus, the mere expectancy that a memory will be used in a future test may determine whether or not sleep benefits consolidation of this memory (see also Reverberi et al., 2020; van Dongen et al., 2012).

Directed forgetting has been investigated not only with the item method but also with the list method. In list-method directed forgetting (LMDF), a forget or remember cue is provided after study of a first list of items, while all participants are asked to remember a subsequently presented second list. On a later memory test, recall of first-list items is typically worse in response to the forget cue (see MacLeod, 1998; Sahakyan et al., 2013). Because, in LMDF, the cues are provided after encoding of the first-list items, the forgetting of first-list items cannot reflect an encoding problem but should be due to impaired retrieval (Pastötter & Bäuml, 2010; Sahakyan & Kelley, 2002). As a result, if differential encoding underlay the present results for IMDF, they may not generalize to LMDF. In contrast, if different expectations elicited by the forget and remember cues mediated the present results, they may generalize to LMDF. Future work is required to address the issue and to uncover whether the observed increase in forgetting rates of TBF items is a general characteristic of directed forgetting or is restricted to IMDF.

Recall rates in the present study were not only analyzed by fitting power functions of time to the recall and recognition scores but were also analyzed using ANOVA. Doing so, results showed a significant interaction between cue and delay in Experiment 1 but no such interaction in Experiment 2, which differs from the results emerging from the power function analysis. The difference in results between methods is not surprising (for further examples, see Carpenter et al., 2008; Wixted, 2022), because ANOVA relies on absolute forgetting rates, whereas power function analysis relies on relative forgetting rates. Wixted (2022) recently provided a number of compelling arguments that indicate that relative, rather than absolute, forgetting rates are theoretically relevant (see also Carpenter et al., 2008; Siler & Benjamin, 2020). Usage of power function analysis to examine time-dependent forgetting and compare the forgetting between item types follows this indication, thus leading to theoretically better motivated conclusions on forgetting over time.

## Conclusions

To our knowledge, this study is the first in the literature to examine time-dependent forgetting of TBF information using IMDF. Results show that both time-dependent forgetting of TBR information and time-dependent forgetting of TBF information follow a power function of time. However, relative forgetting rates are different between the two types of information, with a higher forgetting rate for TBF than for TBR information. The findings are consistent with the view that (mainly) selective rehearsal underlies IMDF. Above all, they demonstrate that the forget cue in IMDF does not only reduce memory shortly after study but also increases forgetting over time.

## Data Availability

Materials and data are available on the Open Science Framework (https://osf.io/j8myp/). Further requests for the data or materials can be sent via email to the corresponding author at anna.nickl@ur.de.

## Footnotes

1 Sometimes, a three-parametric power function of the form *r*(*t*) = *a*(1 + *ct*)^−*b*^, where *c* is an additional scaling parameter, has also been used to describe time-dependent forgetting (e.g., Wickelgren, 1974; Wixted, 2004). In most cases, the two- and three-parameter variants of the function lead to very similar results (see Wixted, 2004), however, which is why, quite often, the simpler two-parameter variant has been employed in the literature (e.g., Bäuml & Trißl, 2022; Rubin & Wenzel, 1996; Wixted & Ebbesen, 1991). We followed this rationale in the present study.

2 Arguably, fits for individual subject data may be preferred over group average data when analyzing time-dependent forgetting (e.g., Anderson & Tweney, 1997). However, to implement this in the present IMDF task, each subject would need to participate in all four retention interval conditions. Doing so would necessarily include some sort of debriefing regarding the test relevance of the TBF information during the first test, and might thus affect performance for this information on the later tests. We therefore preferred group average data, relying on Wixted and Ebbesen’s (1997) finding that not only individual subject data but also group average data are well captured by a power function of time (see also footnote 4 below).

3 The presence of a floor effect in recall of the TBF items would imply that the actual relative forgetting rate of these items would have even been higher than the *b* parameter suggests. The presence of a floor effect thus would not affect the main conclusion from the experiment, i.e., that TBF items show faster forgetting than TBR items.

4 Arithmetically averaging memory scores over participants can produce averaging artifacts, for instance, a group function with mathematical properties that are not representative of the individual participant data (Estes, 1956; Sidman, 1952). One way to evaluate whether or not averaging artifacts were responsible for the present results is to reanalyze the data using geometric averaging (Anderson & Twency, 1997; Wixted & Ebbesen, 1997). Consistent with prior work (e.g., Wixted, 2022; Wixted & Ebbesen, 1997), geometric averaging led to the same pattern of results as arithmetical averaging: estimates of forgetting rate parameter *b* were *.*096 for the TBR items and *.*149 for the TBF items in Experiment 1, and *.*039 for the TBR items and *.*054 for the TBF items in Experiment 2.
